# Heterogeneity in fetal growth velocity

**DOI:** 10.1038/s41598-019-47839-5

**Published:** 2019-08-05

**Authors:** Noriko Sato, Naoyuki Miyasaka

**Affiliations:** 1 Department of Molecular Epidemiology (Epigenetic Epidemiology), Medical Research Institute, Tokyo Medical and Dental University (TMDU), Tokyo, 113-8510 Japan; 2 Comprehensive Reproductive Medicine, Graduate School of Medical and Dental Sciences (Medicine), Tokyo Medical and Dental University (TMDU), Tokyo, 113-8510 Japan

**Keywords:** Intrauterine growth, Intrauterine growth

## Abstract

Fetal growth quality is associated with susceptibility to non-communicable diseases. Fetal size has been conventionally assessed using the averaged growth chart, but fetal growth velocity has recently been attracting attention as another important aspect of fetal development. Since fetal growth velocity may reflect fetal response to various conditions during the developmental process within the maternal constraint, it is reasonable to imagine that there might exist a physiological diversity in growth velocity patterns over time, which has never been explored. We conducted a retrospective cohort study designed to evaluate the heterogeneity of fetal growth velocity in singleton pregnancies in the Japanese population. We leveraged the high frequency of prenatal checkup to collect large numbers of ultrasound measurements of every fetus (N = 801) and computationally analyzed individual changes in growth per week. Latent class trajectory analysis identified three distinct velocity patterns. The variation in growth velocity appeared in the third trimester and corresponded to the differences in neonatal size. This heterogeneity was not simply explained by maternal factors and fetal sex, although those factors had time-varying effects on fetal size. Our findings regarding the heterogeneity in fetal growth velocity will aid in the comprehensive understanding of fetal development quality.

## Introduction

Multiple lines of evidence from epidemiological observations have implicated that the quality of fetal development is linked to risks of common noncommunicable diseases later in life. Developmental Origin of Health and Disease (DOHaD) was conceptualized, which means that the developing conditions *in utero* or in the early phase of life will modify the long-lasting bodily function and physiology^1^. In this context, the problem of an exceptionally high percentage of low-weight-births in Japan has been raised as a serious concern^2,3^. Among these studies or other reports, birthweight or its cross-sectional standardized score of body size has been generally utilized for fetal growth assessment. Although there is no objection that birthweight is indeed a valuable parameter, recent studies have shown that growth velocity provides additional information over knowing fetal size alone^4,5^. Considering the biological aspect, fetal growth velocity reflects the fetal response to various conditions during the developmental process within the maternal constraint^1,6^. Therefore, it is reasonable to imagine that there might exist a physiological diversity in growth velocity patterns over time. Nevertheless, it has not been investigated whether different kinds of heterogeneity exist in fetal growth velocity.

Fortuitously, routine ultrasound scans at prenatal checkups have been conventionally performed relatively frequently in Japan. In some cases, normal ultrasound examinations are conducted on every visit. Therefore, obstetricians empirically observe the heterogeneity in growth curve patterns in clinical settings. Some fetuses show transient growth acceleration followed by deceleration and others show a transient deceleration followed by later acceleration, despite all being within the normal growth range. In light of the DOHaD concept, it may be important to classify such natural variation in fetal growth trajectory. If there is any natural heterogeneity in fetal growth, it is important to characterize the appropriateness of fetal growth by considering physiological factors in addition to pathological risks. Then pre-emptive care from early on can be encouraged upon identification of inappropriate fetal growth based on the DOHaD concept. In reality, however, each mother is exposed to unique combination of multiple risks. Thus, complicated diverse growth variations exist making it difficult to segregate a high-risk population using only birth information. Although it is crucial to identify risk-associated fetal growth trajectories or trajectories prone to disease development, we think that it is also important to know whether there is cryptic (latent) variation in fetal growth in the general population prior to focusing on specific risks or diseases. Moving forward, the difference in the future health that could be generated by the variation in fetal growth velocity needs to be clarified.

Accordingly, we investigated whether there is latent heterogeneity in fetal growth velocity. Prior to this analysis, we also studied the effects of known growth-associated factors^7^, such as fetal sex and maternal attributes, on the fetal growth process.

## Results

### Interaction between time and fetal growth-associated factors

**Figure 1:**
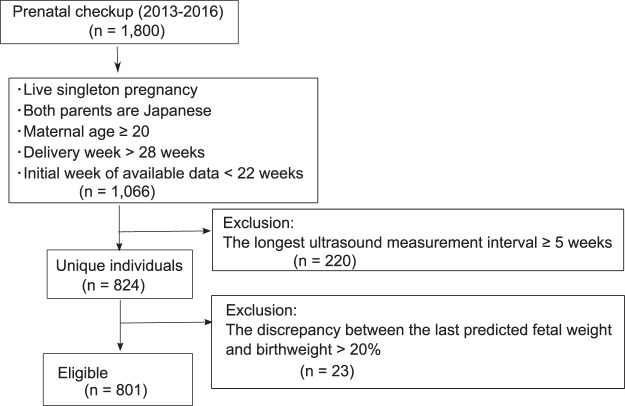
Flow diagram for the pregnant women included in this study.

A total of 801 cases (mothers and fetuses) were eligible for this study (Fig. 1). The estimated fetal weight (EFW) curves of our fetuses were plotted within the range of typical Japanese fetal growth (Supplementary Table S1 and Fig. S1).

**Figure 2:**
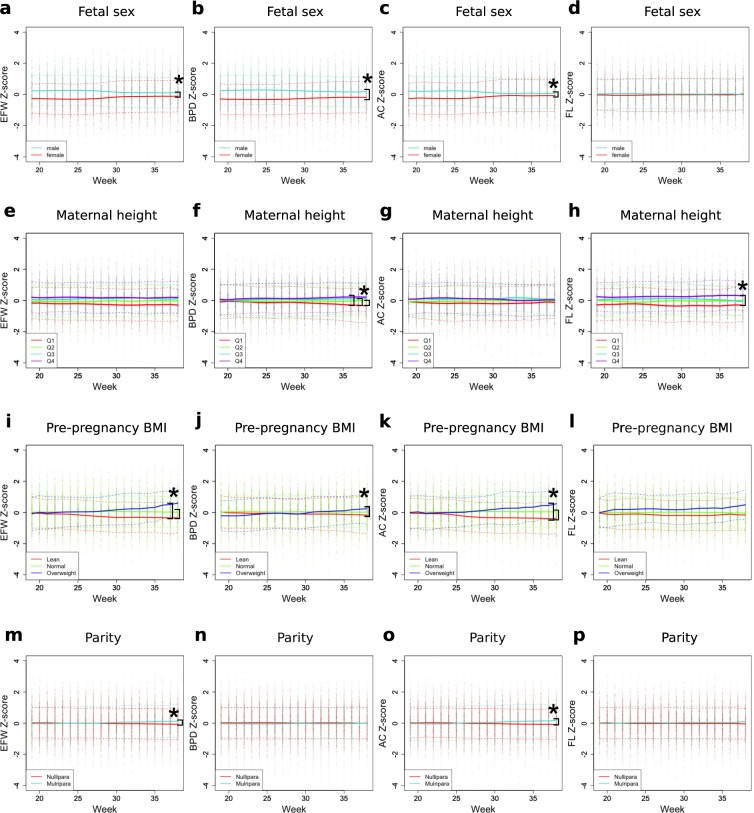
Effects of known associated factors on the trajectories in standardized scores (z-scores) of fetal anthropometric values during gestation. Changes in fetal anthropometric standardized scores (z-scores) of subgroups classified by known factors (such as fetal sex, maternal height, pre-pregnancy BMI, and parity) during gestation are shown. Color codes, showing distinct subgroups for each factor, are indicated in the bottom left. All weekly values (dots) with mean (solid lines) and ±1 SD (dashed lines) of z-scores are shown. Linear mixed models were used to compare groups with the Bonferroni method for multiple testing correction. Asterisk (*) indicates the significant interaction between time and the given factor for each anthropometric value. (Effect size > 0.01 and *p*-value < 2.7 × 10^−4^). EFW, estimated fetal weight; BPD, biparietal diameter; FL, femur length; AC, abdominal circumference; BMI, body mass index.

We first studied whether the known fetal growth-associated factors^7^ (fetal sex, maternal age, maternal height, pre-pregnancy body mass index (BMI), and parity) have week-varying effects. To facilitate detecting a deviation from the population average, the weekly growth parameters (EFW; biparietal diameter, BPD; abdominal circumference, AC; and femur length, FL) were standardized (z-scores) (Fig. 2). Next, association of the known factors with each fetal growth parameter over time was analyzed using multilevel mixed models, by adding the interaction term between the factor and time (see Methods).

Male fetuses (n = 430, 53.7%) were significantly larger than female fetuses in BPD, AC, and EFW at 19 weeks (Fig. 2a–c). However, this sex disparity became significantly smaller in late gestation, indicating the sex-by-time interaction (*P* < 2.0 × 10^−16^).

Maternal height in the first quartile (Q1) was less than 155.8 cm (n = 197, 24.6%); the second quartile (Q2) was from 155.8 to 159.0 cm (n = 209, 26.1%); the third quartile (Q3) was from 159 to 163 cm (n = 185, 23.1%); and the fourth quartile (Q4) was ≥163 cm (n = 205, 25.6%). Q4 showed significantly larger FL z-scores than Q1 at week 19 and this difference became slightly larger over time (*P* = 3.9 × 10^−10^) (Fig. 2h). In addition, the difference between Q1 and other groups gradually enlarged for BPD z-scores, indicating the maternal height-by-time interaction (*P* = 1.8 × 10^−9^ for Q2 and *P < *2.0 × 10^−16^ for Q3 and Q4, Fig. 2f).

Based on pre-pregnancy BMI, mothers were grouped into Lean (BMI < 18.5 kg/m^2^, n = 152, 19%), Normal (18.5 ≤ BMI ≤ 25 kg/m^2^, n = 550, 68.7%), and Overweight (25 kg/m^2^ < BMI, n = 69, 8.6%) groups. At 19 weeks, there was no difference among the three BMI groups; however, AC and EFW z-scores gradually increased in the Overweight, while it gradually decreased in the Lean group over time (Fig. 2i,k). Thus, the significant pre-pregnancy BMI-by-time interaction was shown in AC and EFW z-scores (*P* < 2.0 × 10^−16^).

Parous mothers (n = 269, 33.6%) showed a gradual, but small, increase in AC and EFW z-scores over time (*P* < 2.0 × 10^−16^), although no difference was observed at 19 weeks (Fig. 2m,o).

As for maternal age, the women were divided into three groups: 20 s (n = 152, 19%), 30 s (n = 556, 69.4%), and 40 s (n = 93, 11.6%). There was no statistical significance in the effect of maternal age on fetal growth (Fig. S2).

**Figure 3:**
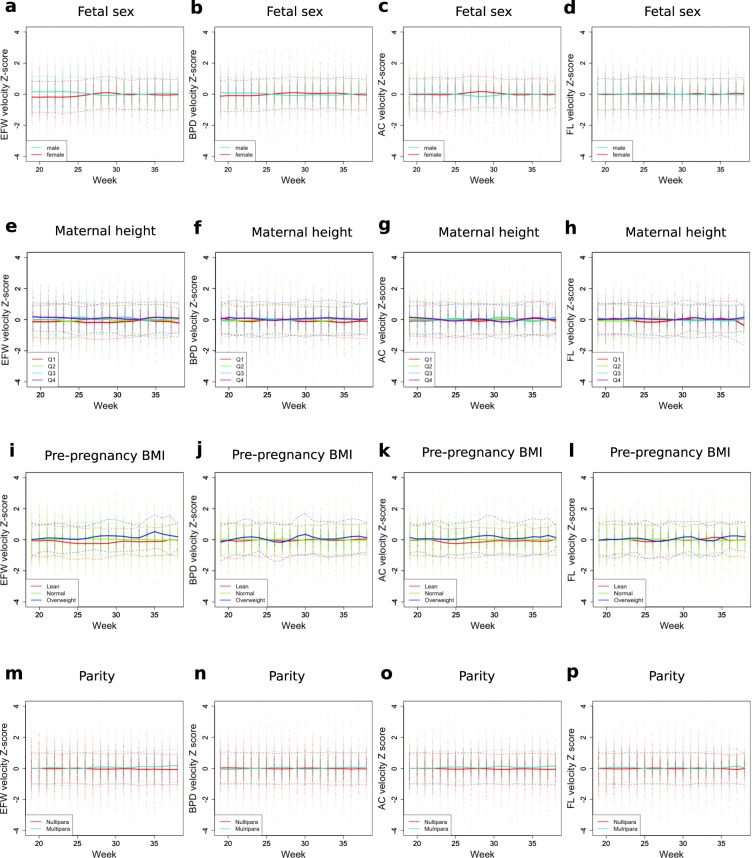
Effects of known associated factors on the trajectories in standardized scores (z-scores) of fetal growth velocities during gestation. Time-varying effects for known factors (such as fetal sex, maternal height, pre-pregnancy BMI, and parity) on fetal growth velocity standardized scores are shown. Color codes, showing distinct subgroups for each factor, are indicated in the bottom left. All weekly values (dots) with mean (solid lines) and ±1 SD (dashed lines) of z-scores are shown. EFW, estimated fetal weight; BPD, biparietal diameter; FL, femur length; AC, abdominal circumference; BMI, body mass index.

To visualize how the above factors are involved in growth velocity over time, the longitudinal velocity z-scores of the subgroups for each category were plotted (Fig. 3). Male EFW velocity z-scores were higher than those of female fetuses until 26 weeks, but this trend reversed during 28–30 weeks (Fig. 3a). It was concordant that the disparity in EFW z-scores was diminished at around 30 weeks (Fig. 2a). In addition, a transient increase of AC velocity z-scores in female fetuses during 27–30 weeks (Fig. 3c) explained the loss of disparity between male and female fetuses in AC z-scores at 30 weeks (Fig. 2c). In a similar manner, week-varying effects of pre-pregnancy BMI and parity on fetal size were related to those week-dependent effects on growth velocity (Figs 2 and 3).

### Latent classes in fetal growth velocity curves

**Figure 4:**
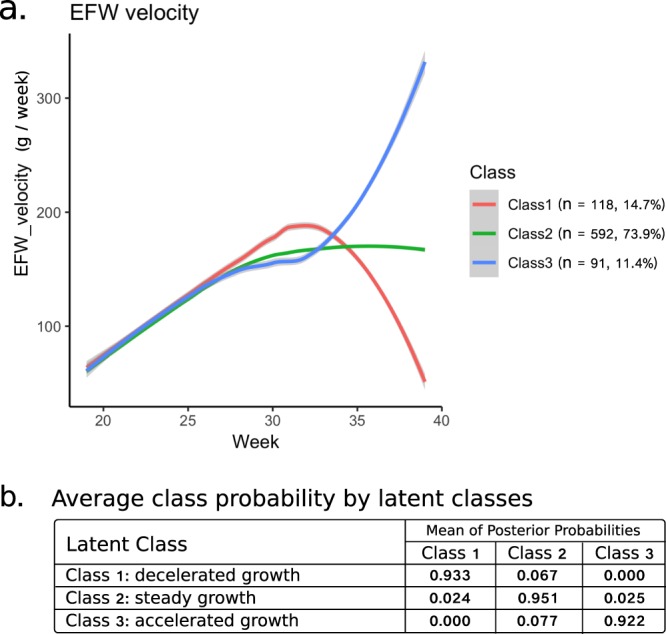
Estimated mean trajectories of estimated fetal weight (EFW) velocity. (**a**) Latent class trajectory analysis identified three distinct trajectories for EFW velocity. Separate colors indicate different classes. (**b**) The levels of the mean posterior probability of class membership for individuals were high (>92%) as shown.

In clinical settings, however, we empirically observe diverse patterns in fetal growth, which are not fully explained by the above factors. To further identify and classify heterogeneity, latent class trajectory analysis was performed for EFW velocity patterns. As a result, three distinct EFW velocity trajectories were identified (Fig. 4). Class 1 (red, n = 118, 14.7%) showed an enhanced acceleration from 25 to 32 weeks and sharp deceleration afterwards (Class of decelerated growth at the end of gestation). Class 2 (green, n = 592, 73.9%) showed monotonic acceleration in fetal growth velocity from 19 to 30 weeks and a peak around 35 weeks and then maintained steady speed afterwards (Class of steady growth). Class 3 (blue, n = 91, 11.4%) showed a slowdown around 25 weeks and a second acceleration from 32 weeks (Class of accelerated growth).

We further investigated the characteristics in each subgroup of trajectories. To examine the relevance between maternal factors and identified classes, the following characteristics were compared but no statistical differences were found among classes: age, pre-pregnancy body size, parity, past poor obstetrics history, complications in the present pregnancy, medically assisted conception and smoking statuses (Table 1). Although the Class 1 and Class 3 mothers showed a higher frequency of noncommunicable disease history than Class 2, it was not statistically significant. In addition, in the sensitivity analysis excluding the mothers with metabolic and hypertensive diseases during gestation (thyroid disease, glucose metabolism disorder, hypertensive disorder of pregnancy), we reproduced a similar result identifying three latent classes (Supplementary Fig. S3). Sensitivity analyses excluding the mothers with preterm (Supplementary Fig. S4) and with pathological risks of low birthweight (smoking during gestation, poor obstetrics history, and complications) (Supplementary Fig. S5) were also performed and similar results were reproduced.

**Table 1:** Maternal characteristics of each trajectory class.

	Class1: decelerated growth (n = 118)	Class2: steady growth (n = 592)	Class3: accelerated growth (n = 91)	Total (n = 801)
Maternal age (year) (mean, (SD))	33.8 (4.6)	33.6 (4.6)	33.5 (4.5)	33.7 (4.6)
Maternal height (cm) (mean, (SD))	158.8 (5.4)	159.1 (5.3)	159.3 (5.0)	159.1 (5.3)
Pre-pregnancy weight (kg) (mean, (SD))	53.0 (8.0)	52.7 (8.5)	54.4 (10.1)	52.9 (8.6)
Pre-pregnancy BMI (kg/m^2^) (mean, (SD))	21.0 (3.0)	20.8 (3.1)	21.4 (3.7)	20.9 (3.2)
Parity (multipara n, (%))	41 (34.7)	193 (32.6)	35 (38.5)	269 (33.6)
Poor obstetrics history^a^ (n, (%))	1 (0.8)	15 (2.5)	1 (1.1)	17 (2.1)
Complications^b^ (n, (%))	3 (2.5)	20 (3.4)	1 (1.1)	24 (3.0)
Disease history^c^ (n, (%))	38 (32.2)	135 (22.8)	26 (28.6)	199 (24.8)
Medically assisted conception (n, (%))	20 (16.9)	100 (16.9)	10 (11.0)	130(16.2)
Smoke (pre-pregnancy) (n, (%))	8 (6.8)	39 (6.6)	6 (6.6)	53 (6.6)
Smoke (during pregnancy) (n, (%))	1 (0.8)	4 (0.7)	0 (0)	5 (0.6)

^a^Poor obstetric history stands for the existence of preterm and/or still birth in the previous delivery. ^b^Complications include hypertensive disorder of pregnancy and preterm labor.

^c^Diseases include asthma, autoimmune disorders, cancer, diabetes, hypertension, hematologic disorders, psychiatric disorders, renal disease, and thyroid disease. BMI, body mass index.

On the other hand, the neonatal characteristics were different among the identified trajectory groups. EFW, BPD, AC and FL velocities, weekly values, and those z-scores in each identified class were plotted (Supplementary Fig. S6). The shape of EFW velocity trajectories resembled that of AC velocity trajectories (Supplementary Fig. S6), indicating that the heterogeneity of AC velocity largely contributed to produce the heterogeneity of EFW velocity. The heterogeneity in velocity and fetal size appeared mainly in the third trimester. The fetuses in Class 1 showed higher z-scores than the others in all the growth measurements (EFW, BPD, AC, FL) during 30–36 weeks (Supplementary Fig. S6). All neonatal sizes (birth weight, birth length, head circumference, and chest circumference) were higher in Class 1 fetuses (*P* < 0.05) (Table 2). The fetuses in Class 3 showed an increase in EFW and AC z-scores after 36 weeks (Supplementary Fig. S6). Neonatal percentile for gestational age (weight, length, and head) was higher in both Class 1 and Class 3 (*P* < 0.05) (Table 2). Consistently, the proportion of heavy for date (HFD) was higher in Class 1 and Class 3 than in Class 2 (*P* = 2.8 × 10^−2^ and *P* = 1.4 × 10^−3^) and the gestational age at delivery was the lowest in Class 3 (*P* = 8.27 × 10^−8^) (Table 2).

**Table 2:** Neonatal characteristics of each trajectory class.

	Class1: decelerated growth (n = 118)	Class2: steady growth (n = 592)	Class3: accelerated growth (n = 91)	Total (n = 801)
Child sex (male N, (%))	66 (55.9)	308 (52.0)	56 (61.5)	430 (53.7)
Gestational age^a^ (weeks), mean (SD)	39.5 (1.2)	39.3 (1.5)	38.4 (1.3)	39.2 (1.4)
Birth weight^b^ (g), mean (SD)	3100 (406)	2978 (414)	2998 (406)	2998 (413)
Birth length^c^ (cm), mean (SD)	50.1 (1.8)	49.4 (2.7)	49.4 (2)	49.5 (2.6)
Head circumference^d^ (cm), mean (SD)	33.8 (1.3)	33.3 (1.3)	33.4 (1.6)	33.4 (1.4)
Chest circumference^e^ (cm), mean (SD)	32.3 (1.5)	31.8 (2.1)	31.7 (1.9)	31.8 (2)
Neonatal weight %tile for gestational age^f^	56.9 (30.1)	49.9 (27.7)	60.3 (28)	52.1 (28.3)
Neonatal length %tile for gestational age^g^	68 (25)	61.1 (25.9)	67.3 (24.6)	62.9 (25.8)
Neonatal head %tile for gestational age^h^	60.1 (26.3)	53.2 (27.2)	58.1 (28.3)	54.8 (27.3)
AGA (n, (%))	89 (75.4)	483 (81.6)	69 (75.8)	641 (80)
HFD^i^ (n, (%))	20 (16.9)	56 (9.5)	20 (22.0)	96 (12)
LFD (n, (%))	5 (4.2)	37 (6.3)	2 (2.2)	44 (5.5)
SGA (n, (%))	4 (3.4)	16 (2.7)	0 (0)	20 (2.5)
Placenta^j^ (g), mean (SD)	585 (116)	557 (109)	584 (106)	564 (111)
Fetus to placenta ratio, mean (SD)	5.4 (0.9)	5.5 (1.1)	5.3 (0.7)	5.5 (1)

Chi-square tests and multiple comparisons of proportion (Ryan’s method) and analysis for variance (ANOVA) were applied for categorical and continuous data, respectively. ^a^Pr(>F) = 8.27 × 10^−8^, ^b^Pr(>F) = 1.29 × 10^−2^, ^c^Pr(>F) = 1.24 × 10^−2^, ^d^Pr(>F) = 7.97 × 10^−3^, ^e^Pr(>F) = 4.96 × 10^−2^, ^f^Pr(>F) = 6.87 × 10^−4^, ^g^Pr(>F) = 6.12 × 10^−3^, ^h^Pr(>F) = 2.13 × 10^−2^, ^i^p = 1.60 × 10^−2^ (Class 1 > Class 2) and p = 6.20 × 10^−4^ (Class 3 > Class 2), ^j^Pr(>F) = 8.53 × 10^−3^. AGA, appropriate for gestational age; HFD, heavy for date; SFD, small for date, SGA, small for gestational age.

In addition to fetal growth velocity, fetal body proportion is an important factor in assessing fetal growth^5^. We further investigated the relationship between growth velocity class and fetal body proportion. The ratios between AC and another reference biometric measurement are commonly used to indicate body proportion^5^. Therefore, we plotted mean trajectories of identified classes for AC/FL or AC/BPD ratio as descriptive analyses (Supplementary Fig. S7a,b). Class 1, but not Class 2 or Class 3 showed a transient increase in the AC/FL ratio between weeks 30 and 36. The AC/BPD ratio increased as gestational age advanced across all the classes, but at the end of gestation, Class 1 and Class 3 diverged reflecting differences in growth velocity patterns between these two classes. Compared to Class 2, both Class 1 and Class 3, on average, exhibited a higher AC/BPD ratio through the entire analysis period. Thus, the trajectory of body proportion was different among identified trajectory groups, mainly during late gestation. We also performed latent class trajectory analysis for AC/FL ratio during gestation and identified four latent patterns (Supplementary Fig. S7c). Although the four distinct patterns showed different curves during gestation, all converged into the similar value towards the end of gestation. Therefore, the AC/FL ratio history (trajectory) was less relevant to neonatal size than the AC/FL ratio at the late gestation or growth velocity trajectories.

It was possible that the higher growth velocity of AC in Class 1 compared to Class 2 and 3 could be caused by some nutritional difference. The maternal weight gain during early-to-mid gestation is partly linked to the nutritional status. Therefore, we explored whether there were any differences among trajectories of identified classes for maternal weight gain as a descriptive analysis. As expected, the maternal weight gain in Class 1 was slightly higher than other classes throughout the 1st and 2nd trimesters (Supplementary Fig. S8).

Additionally, we ruled out the possibility that the inter-observer variability upon ultrasound examinations affected the growth trajectory classification. If this was the case, class distribution would be significantly different among doctors. Therefore, we tested the homogeneity of class-distribution among doctors using the Chi-square test. Three doctors examined over 100 fetuses each among the 801 fetuses analyzed here. As shown in Supplementary Table S2, all the classes of fetuses were similarly distributed among those different doctors.

## Discussion

The time-series fetal size and growth velocity reflects the quality of fetal development. We found variation in the fetal growth velocity patterns in a single ethnic population. Latent class trajectory analysis, using weekly values from 19 to 38 weeks of gestation, identified three distinct velocity patterns. The latent class representing the major group showed acceleration in EFW velocity until 30 weeks and remained steady afterwards. In contrast, two other classes representing minor groups showed enhanced acceleration either during 25–32 weeks or after 32 weeks. These results were in agreement with the obstetricians’ empirical observations.

Maternal demographic characteristics and fetal sex did not show an overall difference among identified trajectories (Tables 1 and 2). Maternal age, height, pre-pregnancy BMI, parity, and fetal sex are well known factors associated with fetal growth^7^. Our study demonstrated that the effects of most factors vary over time. The effect of fetal sex on fetal growth velocity was biphasic. Until 25 weeks of gestation, EFW velocity was higher in male than in female fetuses; however, at 28–29 weeks, it was higher in female than in male fetuses (Fig. 3). Consistently, disparity of EFW weekly size between male and female fetuses became small after 30 weeks (Fig. 2). Interestingly, the FL z-score was not different between males and females throughout the gestation. This result was consistent with many previous reports^8–11^. The EFW velocity in overweight mothers was generally higher, while that in lean mothers was lower (Fig. 3). Accordingly, the EFW size difference caused by pre-pregnancy BMI gradually expanded over time (Fig. 2). The previous study performed ultrasound measurement one time each in the 2nd and 3rd trimesters and showed that maternal BMI was more strongly associated with AC and EFW while maternal height was more strongly associated with FL^9^. We reproduced similar results by performing longitudinal analysis and further found a strong interaction between time and maternal BMI. The EFW velocity and weekly size in parous women slightly increased at the end of gestation (Figs 2 and 3). Conventionally, it is assumed that the effects of those factors are proportional during gestation: for example, fetuses of parous mothers supposedly will have a higher EFW than those of nulliparous mothers by the same proportion at all gestational weeks^12^. However, our study uncovered that growth-associated factors influence fetal development by interacting with time. In spite of time-varying effects on fetal size, those factors could not simply explain the difference among the identified latent trajectory classes.

Neonatal outcomes differed between the identified trajectory classes. The frequency of HFD was higher in both Class 1 and 3. On the other hand, Class 2, the majority group, showed the lowest neonatal size (Table 2). Vasak *et al*. showed that the median birthweight in a population is lower than the centiles for the lowest mortality^13^. These data suggest that the majority of fetuses in a normal condition exhibit some form of maternal constraint on growth^6^. As it is not simple to determine the optimal fetal growth as previously reported^6^, it may be inappropriate to argue which class is better or worse. Additional research is required, in which the children are followed up to determine whether fetal growth velocity patterns are linked to future disease risks.

Grantz *et al*. reported the diversity of the population-averaged fetal growth velocity curves among White, Black, Hispanic, and Asian populations^4^. In that study, participants of diverse ethnicity were randomized to one of four ultrasonography time-schedules and the averaged data derived from four different groups were combined to complement together for obtaining the longitudinal growth curve for each ethnic group. Our approach is a great contrast to Grantz *et al*. We focused on a single ethnic population. The major strength of our study was the analysis of individual growth per week. Since ultrasonography measurements are conventionally conducted frequently in Japan, we were able to estimate the weekly growth for each fetus. This enabled us to find the existence of the large heterogeneity in fetal growth velocity curves, which has never been considered. We detected distinct growth velocity patterns, whose difference was clear in the third trimester. Further, we were able to show the time-varying effects of fetal sex, pre-pregnancy BMI, and parity on developing fetal size, which has never been explored. We think these findings will contribute to a better understanding of the physiological variations, probably produced by the feto-maternal interaction^6^.

The limitations of our study include the retrospective observational design, in which only the second half of the gestation was analyzed. However, it is known that the fetal growth trajectory in the second half of gestation is important in terms of the relevance of the noncommunicable disease risks^6,14,15^. Therefore, our study covered the crucial period for assessing the quality of fetal growth. We identified three main velocity trajectories, none of which was associated with any specific risks, probably because our cohort was not enriched for any special risk population. In most cases, individuals would be exposed to unique combination of multiple risks. This makes it difficult to identify a trajectory relevant to a particular risk, using a general population. Because none of risks accounted for a large percentage of our cohort, aiming to identify a specific risk-associated trajectory is limited given our data. For example, the frequency of smoking during gestation was quite low (less than 1%) in our cohort, although smoking is a well-known factor impairing fetal growth. Although it is indeed preliminarily, we explored how the EFW velocity pattern was affected by smoking and how the velocity trajectory trait influenced the final outcome (Supplementary Fig. S9). Among the three women who smoked during gestation, one woman was overweight and developed gestational diabetes (HbA1c, 9%), which mitigated the effect of smoking for reducing birthweight. The remaining two women were both underweight and showed the similar EFW z-scores at week 20. However, one woman with the minor velocity trajectory trait, Class 1, showed a severe reduction in birthweight. It indicated that the EFW velocity trajectory trait, which itself was not associated with a specific risk, modified the effect size of other risks influencing the birth outcome. We are aware that further specialized investigations to identify risk-associated trajectories or trajectories prone to disease development are crucial. Our approach to seek heterogenous trajectory patterns can be applied to a specialized cohort, including high numbers of women exposed to risks, which will potentially contribute to the identification of risk-associated trajectories.

This study provides an evidence of substantial variation in fetal growth velocity even in a single ethnic population. Further detailed investigation is warranted to establish the appropriate method to judge the quality of fetal growth.

## Methods

### Data source

We performed a retrospective cohort analysis of women delivering at Tokyo Medical and Dental University Hospital from 2013 through 2016 (n = 1800). All the data were anonymously collected from prenatal medical records, which include the following information: maternal height, maternal age, pre-pregnancy BMI, parity, previous obstetrics history (preterm and stillbirth), present pregnancy complication (hypertensive disorder of pregnancy and preterm labor), presence or absence of assisted reproduction technology, history of noncommunicable diseases (asthma, autoimmune disorders, cancer, diabetes mellitus, hematologic disorders, renal diseases, thyroid diseases, hypertension, psychiatric disorders), smoking history, fetal sex, gestational age at delivery, neonatal body size, and placenta weight. Gestational age was determined from the date of the mother’s last menstrual period and confirmed from the measurement of fetal crown-rump length at a first-trimester scan or determined by the date of embryo-transfer in the cases of *in-vitro* fertilization. Typical prenatal check-ups are scheduled at every four weeks until 24 weeks of gestation, every two weeks between 24 and 36 weeks of gestation, and weekly beyond 36 weeks of gestation in Japan^16^. The maternal weight gain at each week was calculated by the weight at each week subtracted by pre-pregnancy weight. In ultrasound examinations, we measured fetal BPD and FL using the linear function and AC using ellipse function for each fetus according to standard techniques suggested by the Japan Society of Ultrasonics in Medicine (JSUM)^17^. The EFW was obtained automatically by the equipment, using the formula proposed by the JSUM (EFW[g] = 1.07 × BPD [cm]^3^ + 0.3 × AC [cm]^2^ × FL [cm]). Ultrasound examinations were performed abdominally by trained obstetricians using 3–5 MHz convex transducers with Voluson E8 (General Electric, MA, USA) ultrasound machines. Neonatal body size percentile for gestational age was calculated using the Japanese neonatal anthropometric chart established by Itabashi *et al*.^18^. The Institutional Review Board approval was obtained from Tokyo Medical and Dental University (approval number M2017-337). Requirement of individual informed consent was waived by the institutional review board because of the retrospective nature of the study and data anonymity of the involved participants. All methods were performed in accordance with the relevant guidelines and regulations.

### Inclusion criteria

The eligibility criteria were as follows: ethnically Japanese singleton women (n = 1515); maternal age above 20 years (n = 1509); gestational age of delivery above 28 weeks (n = 1496), and existing sonogram data before 22 weeks (n = 1066); the longest interval between two consecutive ultrasound measurements was less than five weeks (n = 846); and only one delivery for women with multiple deliveries during the study period (n = 824). Additionally, the last EFW and birthweight (time difference was less than one week) were compared to exclude the large discrepancy (more than 20%) between the sonographic estimation and actual birthweight. A total of 801 cases (women and fetuses) were eligible for this study (Fig. 1).

### Data processing and statistical analysis

The frequency of ultrasound measurements for the analyzed data was 11 ± 1.8 times during gestation for each woman. Using the log-transformed fetal anthropometric measurements, the weekly values were estimated by smoothing splines interpolation using a generalized additive model (“gam” function in “mgcv” R package)^19^. The estimated and observed values were plotted together for each fetus and visually inspected to make sure no overfitting occurred. The fetuses analyzed in our study showed typical Japanese fetal growth curves, confirmed by overlaying the Japanese standard growth chart^17^ (Supplementary Fig. S1 and Table S1). Longitudinal modelling analysis was performed for the period between 19 and 38 weeks of gestational age for the stable estimates (data frequency distribution in Table S1). All the weekly fetal measurements were standardized in the z-score form using the mean and the SD values derived from our study.

For each outcome (z-score trajectory), we fit a multilevel mixed model with each factor and time as fixed effects and variability among individuals as a random effect. The formula of the model was described as follows: EFW z-score ~ fetal sex * time + (1| individual). Tests in multilevel mixed models (“summary.lmerModLmerTest” in “lmerTest” R package)^20^ were conducted to evaluate the significance of factor-by-time interaction for the longitudinal z-scores. A statistical significance of 2.7 × 10^−4^ was used according to the Bonferroni correction because of the multiple testing.

As for the weekly velocity, it was computed as the difference between two consecutive weekly measurement values. To identify patterns of fetal weight growth velocity, latent class trajectory analysis was used (“lcmm” function in “lcmm” R package)^21^. We selected the model in which the weight velocity depends on quartic functions of time because of the lowest Bayesian Information Criteria (BIC) among other polynomial models and did not incorporate any covariates in the model. A linear term for time was used to specify the individual departures from the mean trajectory (random effects). To choose the optimal number of latent classes, the fit of models with different class numbers were compared from one to four. Since the model with three latent classes had the lowest BIC, three latent classes in our study were identified. The best-fit model was EFW velocity ~ poly (time, degree = 4), mixture = ~poly (time, degree = 4), random = ~time, subject = “subject”, ng = 3. On the selected model, we calculated for the posterior probability of each subject to each class and assessed the ability of the model to discriminate between the different classes by inspecting the posterior classification table. For sensitivity analysis, we repeated the latent class models, excluding the mothers with various conditions (metabolic and hypertensive disorders, preterm, or pathological risks). In the descriptive analysis, the maternal or fetal characteristics were compared by the trajectory classes. *P*-value was determined using Chi-square tests with multiple proportions comparison (Ryan procedure) and analysis of variance (ANOVA) for categorical and continuous data, respectively. All analyses were implemented using R (version 3.1.5).

## Supplementary information

Supplementary information

## Data Availability

The datasets generated during and/or analyzed during the current study are available from the corresponding author on reasonable request.
