# Supplementary material for: Heterogeneity in fetal growth velocity
Source: Sci Rep. 2019 Aug 5;9:11304. doi: 10.1038/s41598-019-47839-5 (PMC6683115; doi:10.1038/s41598-019-47839-5)
Supplement: Supplementary file 1 — Supplementary information [file 41598_2019_47839_MOESM1_ESM.pdf]

Heterogeneity in fetal growth velocity

Noriko SATO and Naoyuki MIYASAKA.

Supplementary information

Table S1. Summary statistics of estimated fetal weight (EFW) (n = 801)

Table S2. Heterogeneity detection of growth trajectory is not due to the inter-observer variability

Figure S1. Estimated fetal weight curves of all the fetuses analyzed

Figure S2. Effects of maternal age on the trajectories in standardized scores (z-scores) of fetal anthropometric values during gestation

Figure S3. Estimated mean trajectories of estimated fetal weight (EFW) velocity for the mothers without metabolic and hypertensive diseases during gestation

Figure S4. Estimated mean trajectories of estimated fetal weight (EFW) velocity for the mothers without preterm birth.

Figure S5. Estimated mean trajectories of estimated fetal weight (EFW) velocity for the mothers without pathological risks of low birth weight (smoking during gestation, poor obstetrics history, and complications)

Figure S6. Mean trajectories of identified classes for fetal growth velocity, weekly fetal size, and standardized scores (z-scores) of fetal anthropometric values

Figure S7. The relevance of identified growth velocity class and fetal body proportion

Figure S8. Mean trajectories of identified classes for maternal weight gain.

Figure S9. Preliminary investigation for the involvement of velocity variation in birth outcome.

Table S1. Summary statistics of estimated fetal weight (EFW) (N = 801)

Week of gestational age	Number of missing values	*P* value for normaliy test[a]	Mean of subjects (g)	SD of subjects (g)	Japanese standard (mean, g)
15	617	0.122	73	14	NA[b]
16	508	0.178	107	18	NA[b]
17	388	0.143	147	22	NA[b]
18	287	0.378	196	26	187
19	166	0.293	253	31	247
20	68	0.359	319	37	313
21	0	0.607	391	42	387
22	0	0.829	472	49	469
23	0	0.669	564	56	560
24	0	0.682	666	64	660
25	0	0.411	779	74	771
26	0	0.140	904	85	892
27	0	0.241	1039	95	1023
28	0	0.214	1183	106	1163
29	0	0.135	1335	117	1313
30	1	0.132	1494	128	1470
31	1	0.364	1658	140	1635
32	1	0.393	1825	156	1805
33	2	0.272	1995	172	1980
34	6	0.006	2167	186	2156
35	11	0.012	2339	200	2333
36	20	0.040	2510	216	2507
37	39	0.052	2682	235	2676
38	126	0.383	2850	249	2838
39	312	0.140	3005	247	2989
40	524	0.042	3135	257	3125
41	719	0.834	3263	294	3244

[a]Shapiro-Wilk test; [b]Not described in Okai T. Standard values of ultrasonic measurements in Japanese fetuses. J Med Ultrasonic. 2003;30:J415-440 (Japanese).

Table S2. Heterogeneity detection of growth trajectory is not due to the inter-observer variability

Breakdown of doctors-in-charge and fetal growth velocity classification (n = 801)

	Class 1	Class 2	Class 3
Doctor A	42	158	17
Doctor B	34	177	27
Doctor C	15	81	20
Other doctors	27	176	27

The probability of class distribution was not affected by the observers (doctors), $\chi^2(6) = 11.3$, $p = 0.08$.

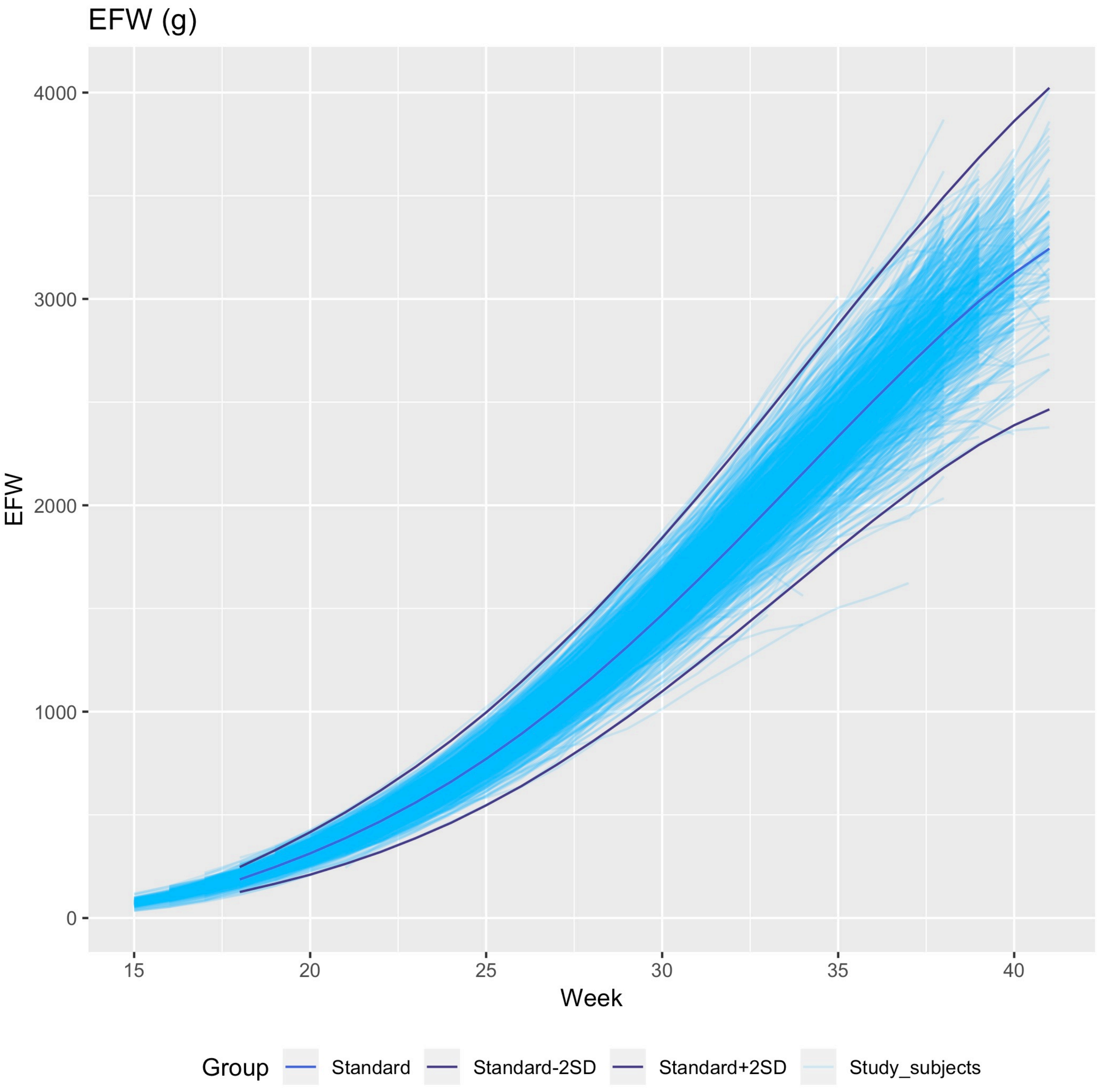

Figure S1. The estimated fetal weight curves of all the subjects in our study

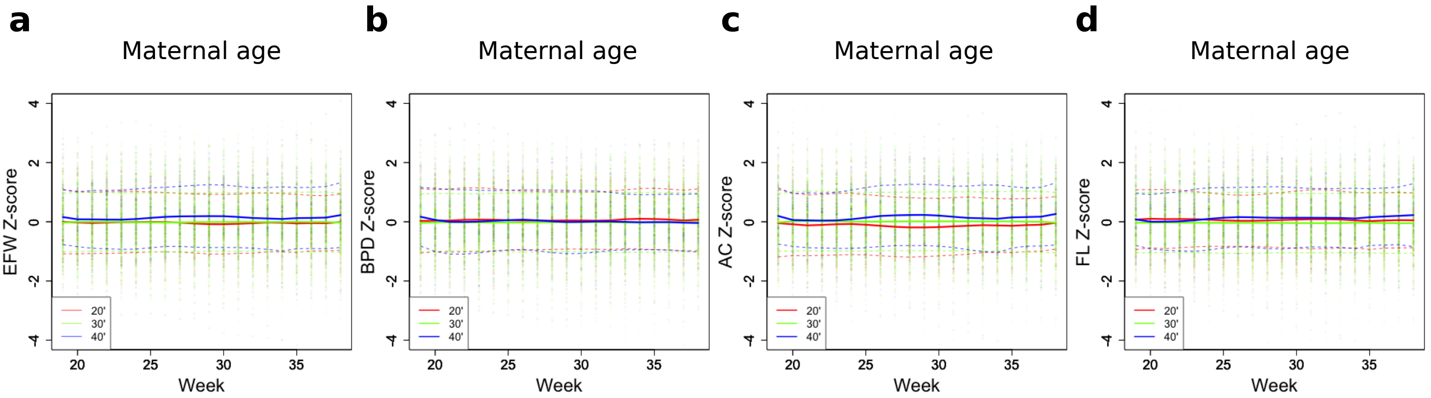

Figure S2. Effects of maternal age on the trajectories in standardized scores (z-scores) of fetal anthropometric values during gestation

Changes in fetal anthropometric standardized scores (z-scores) of subgroups classified by maternal age. Color codes distinct subgroup indicated in the left-bottom. All weakly values (dots) with mean (solid lines) and ± 1 SD (dashed lines) of z-scores are shown. EFW, estimated fetal weight; BPD, biparietal diameter; FL, femur length; AC, abdominal circumference

a. EFW velocity
(no metabolic and hypertensive diseases during gestation)

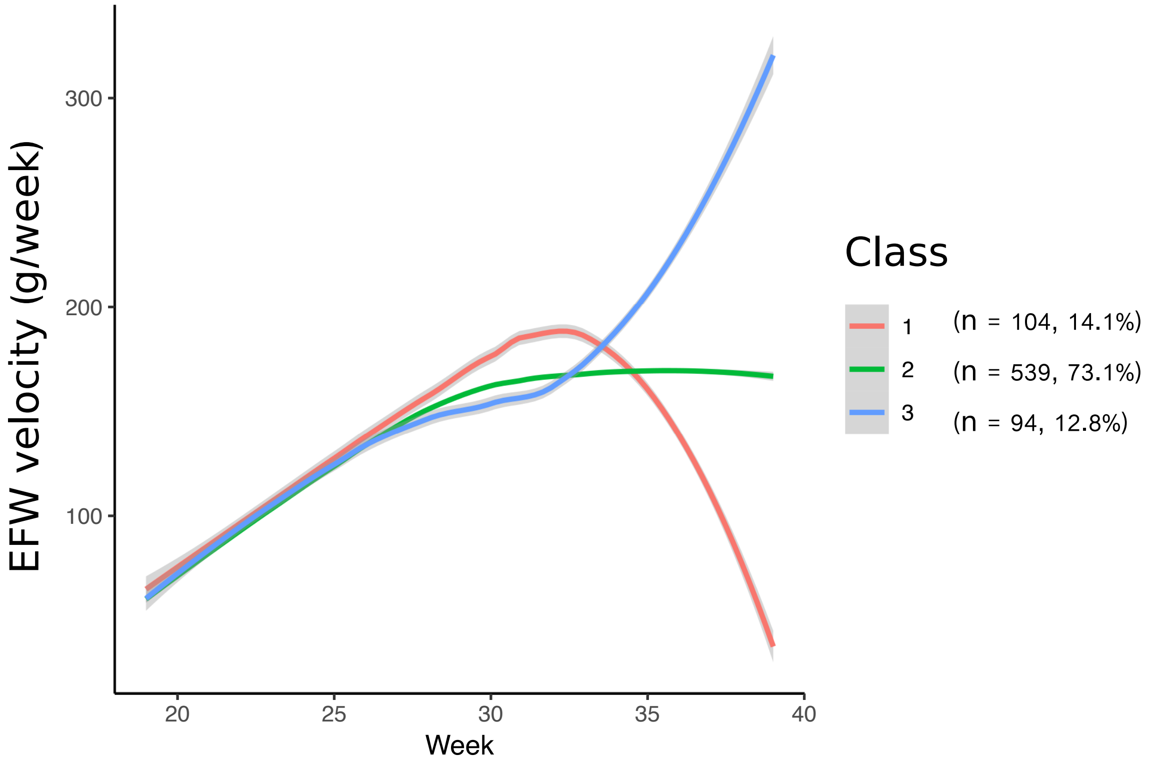

b. Average class probabilities by latent classes

Latent class	Mean of Posterior Probabilities		
	Class 1	Class 2	Class 3
Class 1	0.946	0.055	0.000
Class 2	0.029	0.941	0.030
Class 3	0.001	0.085	0.914

Figure S3. Estimated mean trajectories of estimated fetal weight (EFW) velocity for the mothers without metabolic and hypertensive diseases during gestation (n = 737).

a. Metabolic and hypertensive diseases include thyroid disease, glucose metabolism disorder and hypertensive disorder of pregnancy. Latent class trajectory analysis identified three distinct trajectories for EFW velocity. Separate colors indicate different classes. b. The levels of the mean posterior probability of class membership for individuals are high (> 91%) as shown.

a. EFW velocity
(no preterm birth)

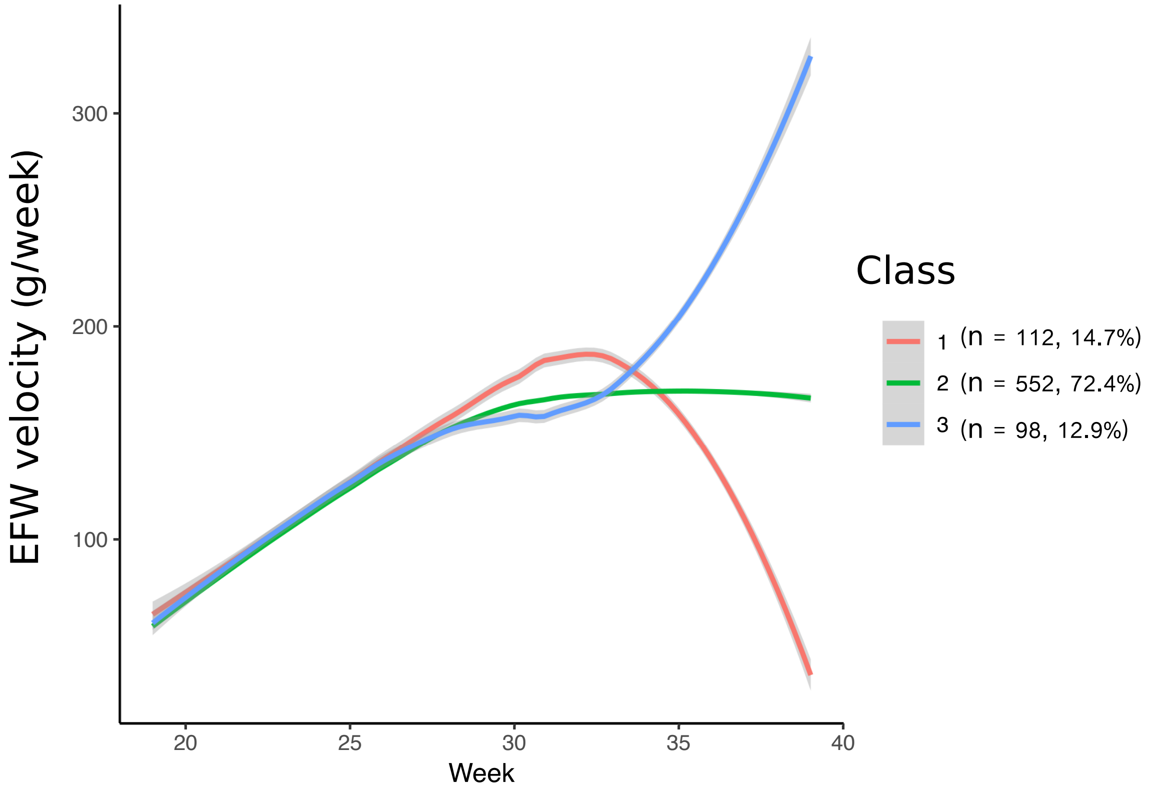

b. Average class probabilities by latent classes

Latent class	Mean of Posterior Probabilities		
	Class 1	Class 2	Class 3
Class 1	0.948	0.052	0.000
Class 2	0.023	0.957	0.020
Class 3	0.000	0.086	0.914

Figure S4. Estimated mean trajectories of estimated fetal weight (EFW) velocity for the mothers without preterm birth (n = 762).

a. Latent class trajectory analysis identified three distinct trajectories for EFW velocity. Separate colors indicate different classes. b. The levels of the mean posterior probability of class membership for individuals are high (> 91%) as shown.

a. EFW velocity
(no smoking, poor obstetrics history and complications)

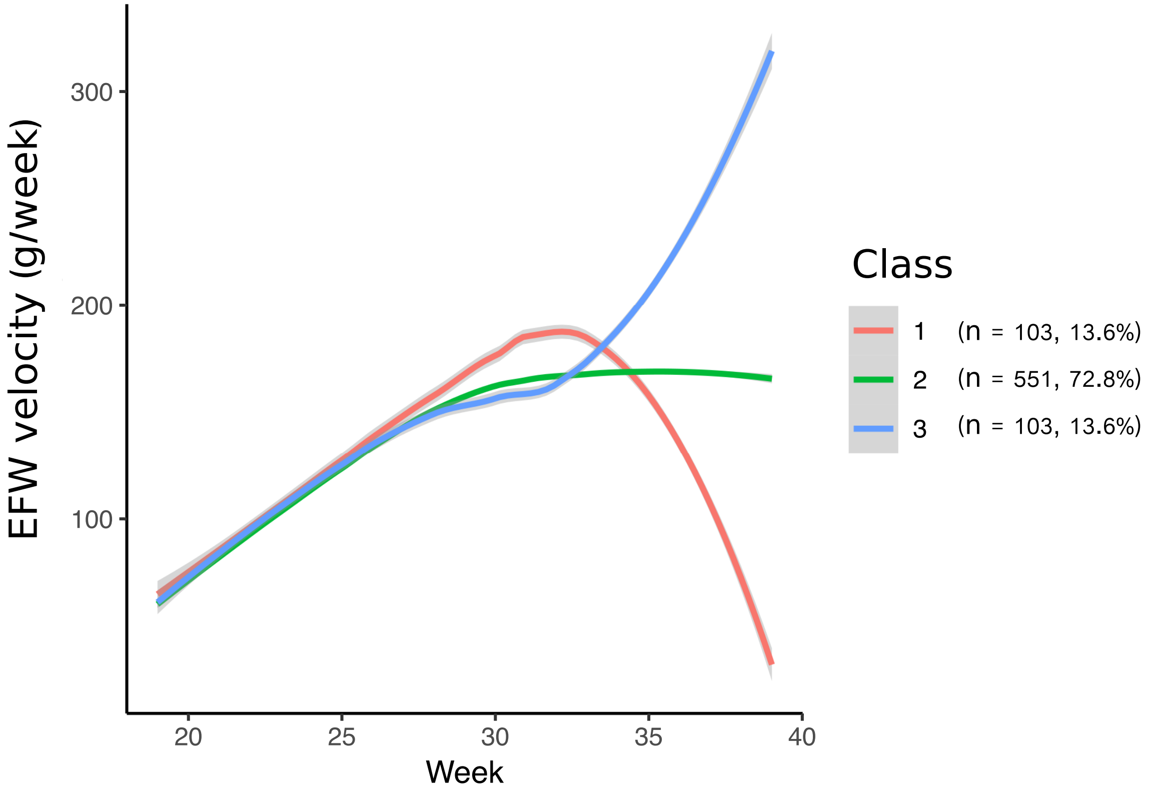

b. Average class probabilities by latent classes

Latent class	Mean of Posterior Probabilities		
	Class 1	Class 2	Class 3
Class 1	0.954	0.046	0.000
Class 2	0.028	0.947	0.026
Class 3	0.001	0.096	0.904

Figure S5. Estimated mean trajectories of estimated fetal weight (EFW) velocity for the mothers without pathological risks of low birth weight (smoking during gestation, poor obstetrics history and complications) (n = 757).

a. Latent class trajectory analysis identified three distinct trajectories for EFW velocity. Separate colors indicate different classes. b. The levels of the mean posterior probability of class membership for individuals are high (> 90%) as shown.

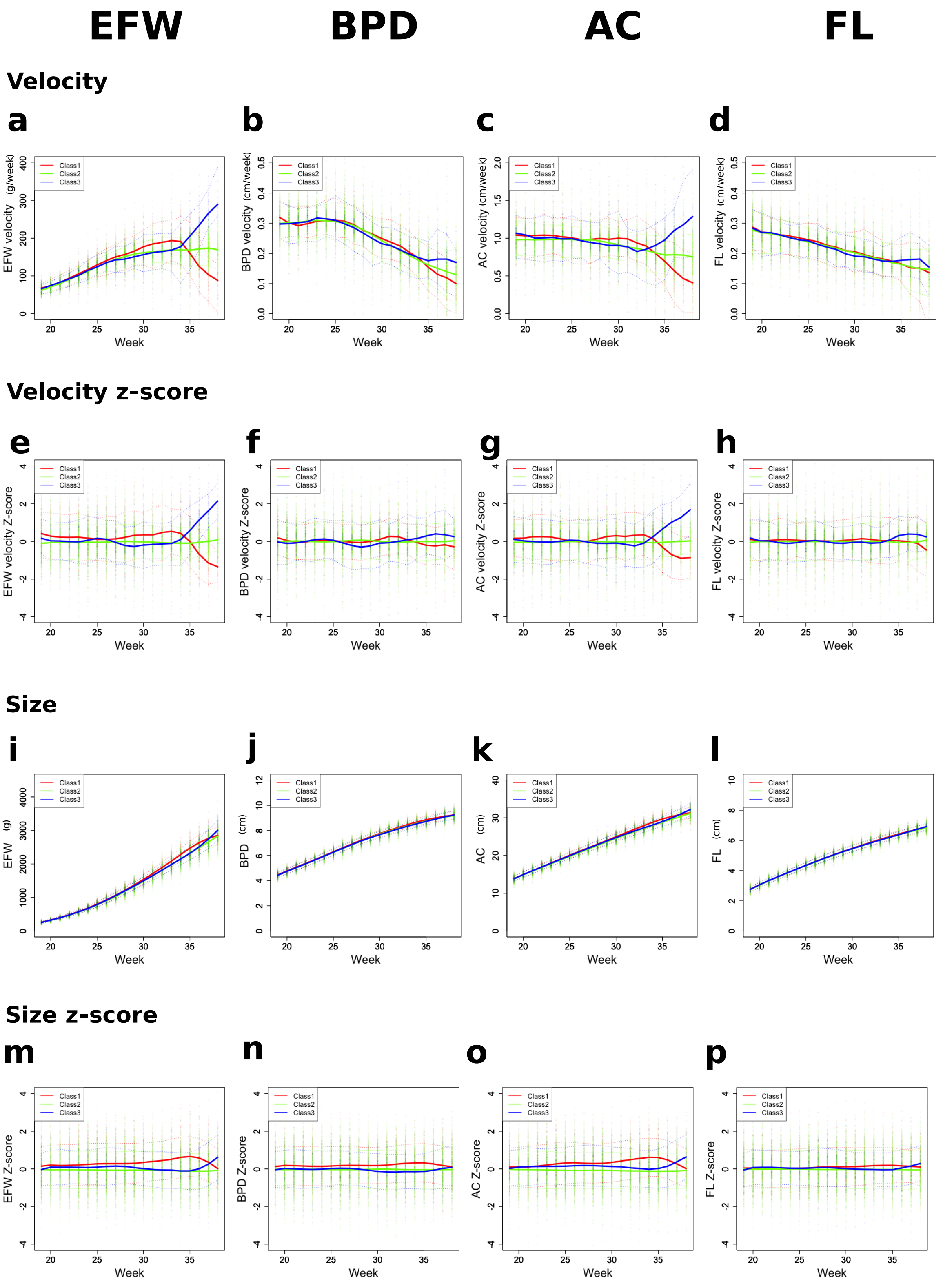

Figure S6. Mean trajectories of identified classes for fetal growth velocity, weekly fetal size, and their standardized scores (z-scores) of fetal anthropometric values

Color codes identified class indicated in the top-left. All weekly values (dots) with median (solid lines) and $10^{th}/90^{th}$ (dashed lines) for **a-d**, with mean (solid lines) and ± 1 SD (dashed lines) for **e-p** are shown. EFW, estimated fetal weight; BPD, biparietal diameter; FL, femur length; AC, abdominal circumference

a. b

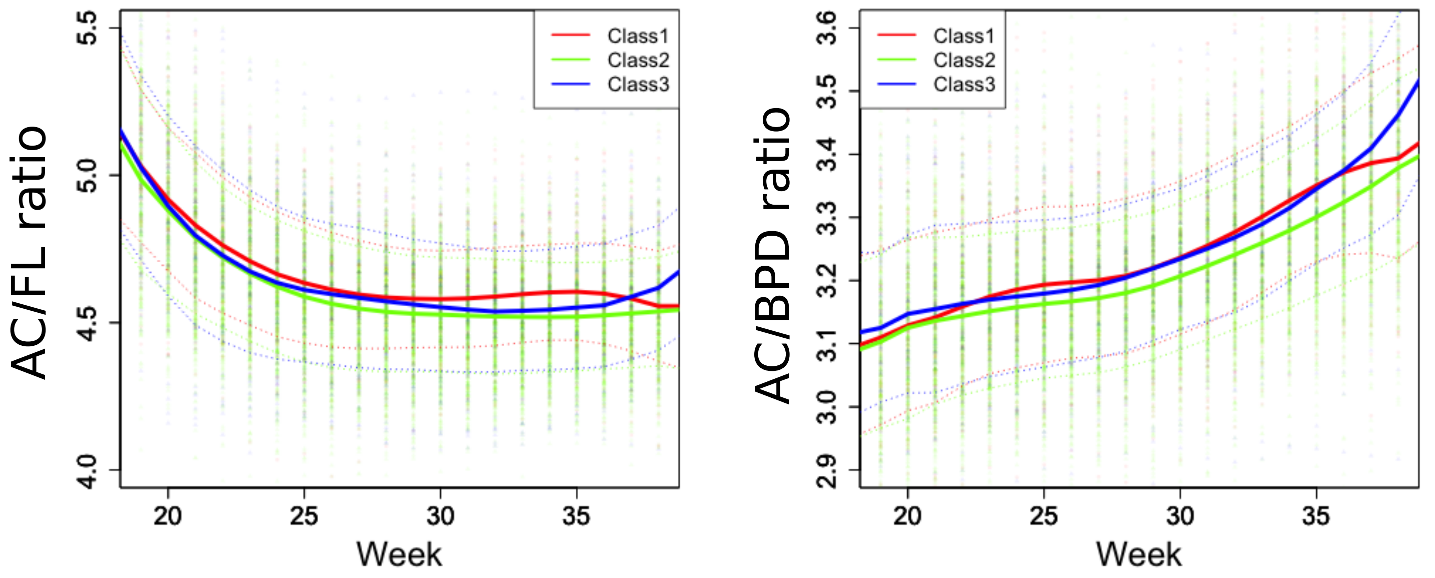

c.

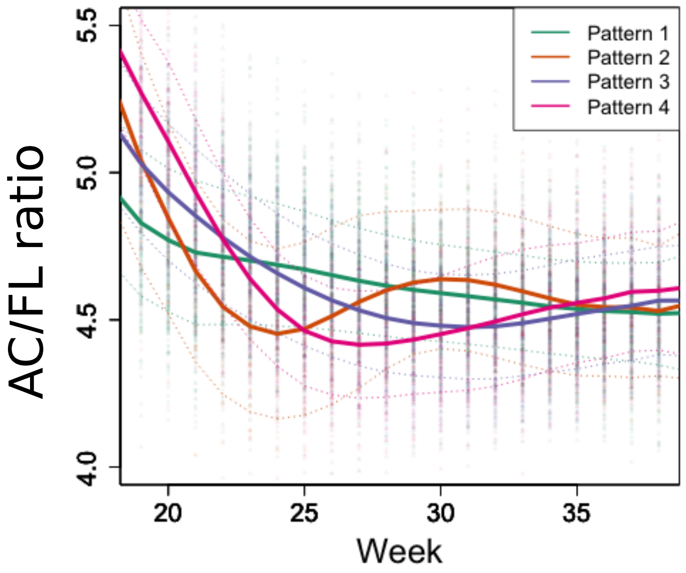

Pattern 1 (n = 330, 41.2%)
Pattern 2 (n = 83, 10.4%)
Pattern 3 (n = 266, 33.2%)
Pattern 4 (n = 122, 15.2%)

Average class probability by latent class

	Mean of Posterior Probabilities			
	Pattern 1	Pattern 2	Pattern 3	Pattern 4
Pattern 1	0.969	0.008	0.022	0.001
Pattern 2	0.018	0.959	0.018	0.005
Pattern 3	0.030	0.009	0.955	0.007
Pattern 4	0.003	0.006	0.026	0.966

Proportion of velocity classes in each AC/FL trajectory pattern

	Class 1	Class 2	Class 3
Pattern 1	0.142	0.745	0.112
Pattern 2	0.217	0.639	0.145
Pattern 3	0.124	0.759	0.117
Pattern 4	0.164	0.764	0.090

Figure S7. The relevance of identified growth velocity class and fetal body proportion. Mean trajectory of identified velocity trajectory classes for AC/FL ratio (a) and AC/BPD ratio (b). Mean trajectory of identified AC/FL ratio trajectory patterns by latent class trajectory analysis of AC/FL ratio (c). All weekly values (dot) with mean (solid lines) and ± 1 SD (dashed lines). Color codes identified class or pattern indicated in the top. BPD, biparietal diameter; FL, femur length; AC, abdominal circumference.

a.

b.

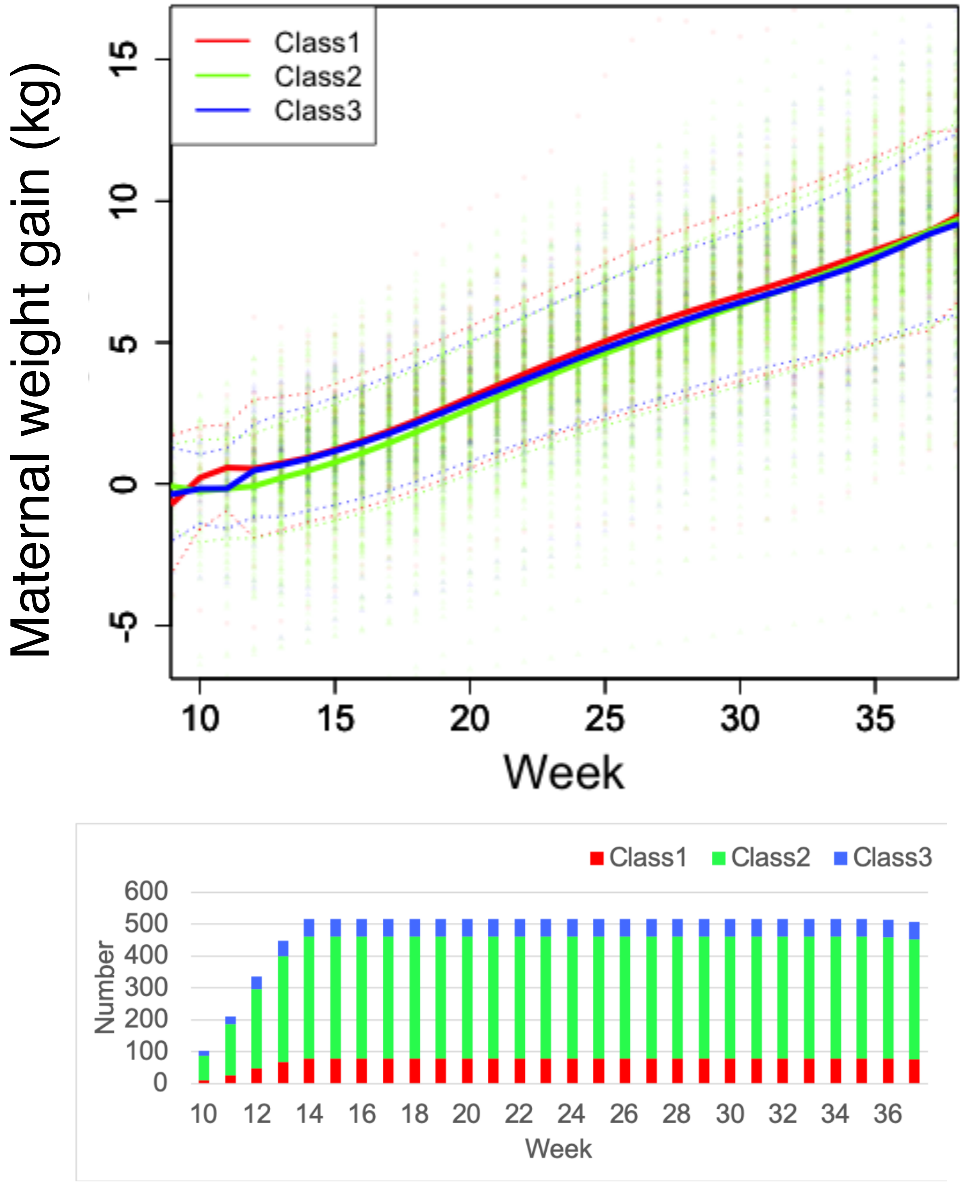

Figure S8. Mean trajectories of identified classes for maternal weight gain. a. Mean trajectory of identified velocity trajectory classes for maternal weight gain. b. Sample number each week shown in a stacked bar chart. Color codes identified class indicated in the top.

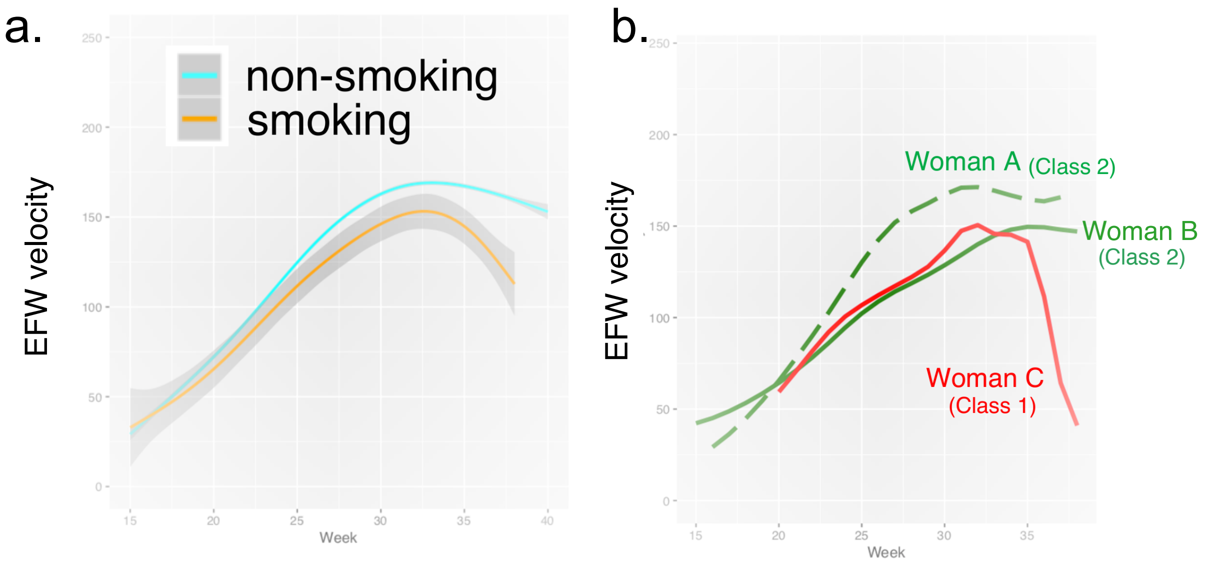

c.

Woman	Week of delivery	Maternal characteristics			Child characteristics				
		GDM or No	Age (y)	Pre-pregnancy BMI (kg/m^2)	EFW z-score at week20	Birth weight (g)	BWGA z-score	Ponderal index	NICU admission
A	38.8	GDM	33	31.2	-1.00	3380	1.39	2.55	Yes
B	39.4	No	29	15.8	-0.15	2866	-0.12	2.51	No
C	39	No	30	18.2	-0.13	2192	-2.40	1.86	Yes

Figure S9. Preliminary investigation for the involvement of velocity variation in birth outcome.
Among term vaginal deliveries (n = 495), three women smoked during gestation. a. EFW velocity of smoking and non-smoking groups. b. EFW velocity curve of each woman in the smoking group. c. The characteristics of women and their children in the smoking group. GDM, gestational diabetes mellitus; BMI, body mass index; EFW, estimated fetal weight; BWGA, birth weight for gestational age; NICU, Neonatal intensive care unit
